# Healthcare-Seeking Behavioural Practices Among Pregnant Women in Odeda Local Government Area, Ogun State, Nigeria

**DOI:** 10.1155/jnme/9956172

**Published:** 2025-11-25

**Authors:** Y. O. Adebayo, O. B. Akinsanya, O. T. Lasabi, R. O. Adeoye, A. M. Ademola

**Affiliations:** ^1^Nutrition and Dietetics Department, Federal University of Agriculture, Abeokuta, Ogun State, Nigeria; ^2^Department of Food Science and Technology, Nutrition and Dietetics Unit, Bells University of Technology, PMB 1015, Ota, Ogun State, Nigeria; ^3^Dietetics and Human Nutrition, School of Health Sciences, University of KwaZulu-Natal, Private Bag X01, Scottsville, Pietermaritzburg 3209, South Africa; ^4^Institute of Food Security, Environmental Resources and Agricultural Research, Federal University of Agriculture, Abeokuta, Nigeria; ^5^Food Science and Technology Department, Enugu State University of Science and Technology, Agbani, Enugu, Nigeria

**Keywords:** healthcare, maternal care, pregnancy

## Abstract

**Background:**

Maternal mortality is a global health concern, particularly in developing nations, such as Nigeria. Pregnant women face health challenges and complications, necessitating prompt attention to understand healthcare-seeking behaviours to improve maternal health outcomes.

**Objective:**

To assess the healthcare-seeking behavioural practices among pregnant women in Odeda Local Government Area, Ogun State, Nigeria.

**Methodology:**

A descriptive cross-sectional study was conducted among 200 pregnant women attending antenatal clinics at five selected primary healthcare centres in the Odeda Local Government Area. Data were collected using a structured questionnaire and analysed using the Statistical Package for the Social Sciences (SPSS) Version 20.0 for descriptive statistics, such as frequencies, percentages and chi-square tests to explore the association between sociodemographic characteristics and healthcare-seeking behaviours.

**Results:**

Most respondents (94%) were married, with an average age of 27 years. Approximately 48.5% had a secondary education, while 42.5% had tertiary education. A significant number of respondents (78%) were knowledgeable about danger signs during pregnancy, such as vaginal bleeding, high fever and reduced foetal movement. Regarding healthcare-seeking practices, 54.5% of the respondents sought healthcare at hospitals or clinics when they experienced pregnancy complications. However, 18% resorted to praying first, and 22% informed their husbands before taking further action. The decision to seek care was influenced primarily by husbands in 53% of the cases.

**Conclusion:**

Sociodemographic factors, such as age of the respondents, gestational age and marital status, influence healthcare-seeking behaviour among pregnant women in the Odeda Local Government Area. Although there is a high knowledge of pregnancy danger signs, timely healthcare actions are needed to reduce maternal mortality rates.

## 1. Introduction

Healthcare-seeking behaviour can be understood as the actions individuals take to address perceived health problems, including the decision of when and where to seek healthcare services [1]. This behaviour is especially important during pregnancy, a period characterized by significant physiological and emotional changes, which can affect both maternal and infant health [2].

Pregnancy is a period of transition with significant physical and emotional changes. Even in uncomplicated pregnancies, these changes can affect the quality of life of pregnant women and maternal and infant health (pregnancy monitoring, pregnancy outcomes, maternal postpartum health and the psychomotor development of the infant) [1].

Regular attendance at antenatal care (ANC) clinics has been found to play an important role in the early discovery of pregnancy-related complications through monitoring of their vital statistics comprising weight measurement, blood pressure, routine vaccinations, abdominal examinations and anaemia management [3]. Women with regular attendance at antenatal clinics are bound to value the significance of appropriate medical interventions and seek healthcare promptly in the event of any form of pregnancy-related complaints.

Maternal mortality, the death of a woman during pregnancy or within 42 days of termination of pregnancy, remains a pressing issue globally. Approximately 800 women die daily from preventable causes related to pregnancy and childbirth, with 99% of these deaths occurring in developing countries [4], particularly in rural and underserved areas [5]. Nigeria has one of the highest maternal mortality rates globally, and it contributes significantly to the overall global burden of maternal deaths accounting for over one-quarter (28.3%) of all estimated global maternal deaths with approximately 8200 maternal deaths [6]. Estimates indicate that Nigeria alone accounts for a substantial portion of global maternal deaths such that it is ranked among the top three in Africa with the highest maternal mortality rates as the latest data indicate a maternal mortality ratio of 1047 per 100,000 live births [7]. Key contributors to these deaths include haemorrhage, hypertensive disorders, obstructed labour and unsafe abortions, which can often be mitigated with appropriate ANC [8, 9].

The provision of ANC is essential to improving maternal health outcomes. It allows for early identification of risk factors, management of complications and preparation for childbirth [10]. Driving factors underlying low access and utilization were summarized under six key subthemes: women's lack of autonomy and decision-making power, sociocultural barriers to attending health facilities, low perceived need and benefit of pregnancy care, economic barriers to access, physical access barriers and perceived quality of care at facilities [11]. The goal of ANC is to prepare mothers for birth and parenthood and prevent, detect, alleviate or manage health problems during pregnancy that affect mothers and babies [12].

Many research studies have been carried out on other factors that influence the healthcare-seeking behavioural practices of pregnant women, such as poor healthcare facilities, poor health-seeking practices, cost of healthcare services, social structures, health beliefs, education, occupation, maternal age and distance to health facilities [8]. However, challenges, such as insecurity in certain regions, fluctuating economic conditions and inconsistencies in healthcare funding and policy implementation, continue to complicate access to maternal health services in Nigeria [13, 14]. In addition, the widespread dissemination of misinformation—particularly through social media—can negatively influence health-seeking behaviours among pregnant women [15]. Rural women and adolescent mothers also encounter unique and often underexplored barriers [16]. Thus, the need for continuous understanding of the healthcare-seeking behavioural practices among women to generate insights can further improve maternal health outcomes in Nigeria. Understanding healthcare-seeking behaviour among pregnant women is crucial for addressing these challenges and improving maternal health in Nigeria.

## 2. Methodology

### 2.1. Study Area

The study was conducted in antenatal clinics of primary healthcare centres (PHCs) in the Odeda Local Government Area (LGA) of Ogun State. The LGA of study is one of the local governments in the state with its headquarters in Odeda town located at Abeokuta-Ibadan Road. It is comprised of ten [10] wards bounded by Bakatare, a small village close to Oyo State, and Alogi, an important urban centre that bounds the LGA from Abeokuta South. It is a rural area strategically located in the Egba division of Ogun State with a strong agricultural base. It shares boundaries with 2 other LGAs in the state: Abeokuta North and Obafemi Owode as well as Oyo State to the north. There are sixteen [16] PHCs in the LGA [17].

### 2.2. Study Design

A descriptive cross-sectional design was adopted for this study. It is comprised of 200 respondents attending the PHCs in Odeda LGA of Ogun State.

### 2.3. Eligibility Criteria

• Inclusion Criteria: Pregnant women attending antenatal clinics at the selected PHCs at the time of the study were included.
• Exclusion Criteria: Pregnant women who were unwilling to participate or did not give consent as well as those with chronic medical conditions, speech impairments or hearing impairments that could interfere with their ability to respond accurately to the instrument of study were excluded from the study.

### 2.4. Sample Size Determination

The sample size was determined using Fisher's formula [18]:

(1) $$\begin{array}{c} {N=Z}^{2}\frac{\text{pq}}{d^{2}}, \end{array}$$

where *N* = minimum sample required, *Z* = standard normal deviate at 95% confidence level = 1.96 from the normal distribution table, *d* = desired precision = 5% = 0.05, *p* = prevalence of healthcare-seeking of pregnant women attending antenatal clinic = 10.5% (0.105) [19].

(2) $$\begin{array}{c} q=1-p,\\ N=\frac{{\left(1.96\right)}^{2}\times 0.105\times 0.89}{{\left(0.05\right)}^{2}}=\frac{384\times 0.0979}{0.0025}=150.37,\\ 10\%\text{ of }150.37=15,\\ 150.37+15=165.37, \end{array}$$

10% of the calculated sample size was added and approximated to 200 respondents to account for the missing questionnaire and improve representation.

### 2.5. Sampling Procedure

A multistage sampling procedure was used for this study.

  Stage 1: A simple random sampling method was used to select five [5] from the sixteen [16] PHCs in the Odeda LGA of study. The selected PHCs are as follows:
• Obantoko PHC
• Osiele PHC
• Olodo PHC
• Camp (Aaye) PHC
• Odeda PHC
  Stage 2: A systematic sampling method was used to select 40 pregnant women from each of the five PHCs from the antenatal register to make up the 200 respondents of the study. The antenatal register served as the sampling frame.

### 2.6. Informed Consent

Informed consent was taken from all respondents before data collection. Informed consent was written on the questionnaire and included a verbal explanation describing the purpose of the study, a procedure for maintaining confidentiality and the respondents' right to refuse to participate. Also, before the study, an approval was sought and obtained from the Nutrition and Dietetics Department of the Federal University of Agriculture, Abeokuta, Ogun State, Nigeria. Ethical clearance was obtained from the Health Research and Ethics Committee, Department of Planning, Research and Statistics of the Federal Medical Centre, Abeokuta (FMCA/470/HREC/01/2022/05).

### 2.7. Data Collection

An interviewer-administered questionnaire divided into 3 sections was used to elicit relevant information from the respondents.  Section 1 provided information on the age, marital status, religion, education level, occupation of respondents, occupation of respondent's husband and type of family of the respondents.  Section 2 adopted the Maternal Serum Screening (MSS) Prenatal Screening Guide to obtain data on knowledge of danger signs and identify the factors associated with them [20].  Section 3 captured the healthcare behavioural practices of the respondents during pregnancy and their influencing factors, such as the preferred place of delivery, persons accompanied to a health facility for obstetric care, final decision-making regarding the place of birth and the total number of pregnancies.

### 2.8. Data Analysis

Descriptive statistics, such as frequency, percentage and mean, were used to describe relevant variables. Inferential analysis, such as chi-square test and correlation, was used to interpret variables using the Statistical Package for the Social Sciences (SPSS) Version 20.0. A *p*-value of < 0.05 was taken as significant.

## 3. Results

Table 1 shows the sociodemographic and socioeconomic characteristics of the pregnant women. The age of women ranged from 18 to 49 years with a mean age of 27.03 ± 5.58 years. They were mostly married (94.0%) with 41.5% in the second trimester of pregnancy. Most (44.5%) of the women were traders. The occupation of the spouse indicated 34.5% as businessmen. The education level of the pregnant women in the study showed that 48.5% of respondents had secondary education, while 5% had no formal education. Most (89.5%) were from nuclear families with 10.5% from polygamous homes.

Table 2 shows the results of the maternal health and ANC indicators among the respondents. The majority of respondents (74.5%) planned their pregnancy, while 25.5% did not. Approximately half of the respondents (57%) began ANC in the first trimester, 38% in the second trimester and 5% in the third trimester. According to the findings, 86% of respondents have never had a stillbirth or miscarriage, whereas 14% have had a stillbirth or miscarriage. The vast majority of responders (92%) report no health issues or medical concerns. The majority of responders (78%) are aware that warning indications during pregnancy might be harmful to both the kid and the mother. The study also finds that 78% of respondents learn about danger indicators from health/medical workers and 3.5% learn about them from friends.

Table 3 displays the pregnant women's knowledge of danger signs of pregnancy. It was discovered that the majority of women, 86% and 81%, are aware that weakness and high fever are both risk indications in pregnancy. Seventy-seven per cent were aware of persistent abdominal pain, a significant reduction in foetal movement (71.5%), vaginal bleeding and swelling of the feet and face (68.5%), while 54% were aware of breathing trouble and convulsion (44%) as danger signs in pregnancy.

Table 4 shows the results of respondents' healthcare-seeking behaviour. Apart from being pregnant, the majority of respondents (92%) do not experience any health problems or ill health. It was also revealed that 4.0% of the respondents were asthmatic, while 0.5% had diabetes, and 0.5% had hypertension, with the majority (93.5%) not having any bad health/health concern other than being pregnant. The study also revealed that when experiencing pregnancy health problems/complications, a high proportion of respondents (54.5%) seek hospital/healthcare, and 2.2% contact friends and family. The study also revealed that about half of the respondents (53%) were influenced by their husbands in seeking healthcare services, and 2.0% of the respondents were influenced by neighbours. The majority of the respondents (63%) enrolled at PHCs alone, while 37% registered at PHCs in conjunction with other healthcare facilities, such as religious homes, general hospitals, and teaching hospitals.

Table 5 depicts the relationship between sociodemographic variables and pregnancy health concerns. Apart from being pregnant, the study assesses the observation level of perceived ill health. It was discovered that there is a significant relationship between health problems and age (*p* = 0.03), and gestational age (*p* = 0.017), but no significant relationship with marital status (0.42), religion (0.57) and education level (0.42).

Table 6 shows the relationship between sociodemographic and healthcare-seeking practice (reason for choosing PHC). The result revealed that there was no significant relationship between age (*p* = 0.25), religion (*p* = 0.981), occupation of the spouse (*p* = 0.72), educational level (*p* = 0.78) and the choice of the healthcare system. However, among the respondents, a significant relationship was observed between marital status and choice of healthcare centre (*p* < 0.001).

Table 7 shows the relationship between sociodemographic and healthcare-seeking practice (healthcare-seeking influence). The results show that there is no significant relationship between age (*p* = 0.185), educational level (*p* = 0.987) and factors influencing the choice of healthcare system. However, a significant relationship was observed between marital status (0.004), religion (0.000), occupation (0.008) and factors influencing the respondents' choice of healthcare centre (husband, self, neighbour, friends and relative).

Table 8 shows the relationship between sociodemographics and healthcare-seeking practice (report of port of call for healthcare). The result showed that there is no significant relationship between age (*p* = 0.684), religion (0.639), occupation of the spouse (0.72), educational level (0.987) and report of port of call for healthcare. However, among the respondents, a significant relationship was observed between religion (0.023), occupation of spouse (0.02) and report of port of call for the healthcare centre.

## 4. Discussion

This study assessed healthcare-seeking behavioural practices among pregnant women in the Odeda LGA of Ogun State. The research identified how pregnant women in Odeda LGA of Ogun State access healthcare services during pregnancy, their knowledge of danger signs and the factors influencing their healthcare-seeking behaviour.

The age of women is a crucial factor in determining the health outcomes of both the mother and the child. This study revealed that about 45% of women were in the 24–29 age group which falls within the range of an ideal age group for pregnancy which is 20–30 years of age and 12% of women were within the age group above 35 years of age. This is an indication of low teenage pregnancy among the respondents as few of them were below the ideal age group for pregnancy corroborated by their marital status with less than 5% single among the respondents of the study. This is similar to the findings of Das et al. [21] which revealed that a high percentage of the women were in the 25–30 age group and few were in the age group above 35, while Egbuniwe et al. [19] also revealed similar findings. However, in the study conducted by Nwachukwu et al. [22] in Delta State, Nigeria, it was observed that the majority of the respondents were between the age ranges of 28 and 33 years. The low teenage pregnancy pattern observed in all these studies could be because the studies were not conducted in certain regions of the country particularly the northern part where there are higher rates of adolescent pregnancy as early marriages, which increases the risk of teenage pregnancy, is seen as part of the cultural norms. Therefore, the percentage of childbearing adolescent women highly varies by region depending on cultural, religious, political, economic and other factors [23].

The findings of this study reported that most of the respondents had secondary and tertiary education, while others attained low educational status. Studies indicate that women with lower levels of education are less likely to attend ANC, utilize maternal healthcare services, and seek help for pregnancy-related complications. This can lead to limited knowledge about pregnancy, childbirth and maternal healthcare, hindering women's ability to make informed decisions [23]. However, Das et al. [21] revealed a high level of respondents with a primary level of education. The study also shows that most of the respondents are from monogamous families, which is similar to the study conducted by Gopalakrishnan et al. [5] in India, which reveals that the majority of respondents are from nuclear families.

The study revealed that the majority of the respondents were aware of the effect of danger signs on both the child and the mother and that most of the respondents got knowledge about danger signs from health/medical personnel. However, this proportion is similar to that reported by Das et al. [21] and Nwachukwu et al. [22] indicating that the majority of respondents got knowledge from health/medical personnel. The majority of the respondents were aware that vaginal bleeding and swelling of the feet and face are danger signs in pregnancy. Other danger signs indicated by the respondents comprised of marked decrease in foetal movement, blurred vision with high fever, persistent abdominal pain, high fever, weakness, convulsions and breathing difficulties. This contradicts the results of the study in Ethiopia by Abdurashid et al. [24] which indicated that most of the respondents do not recognize vaginal bleeding, swollen face and feet, blurred vision, reduced foetal movement and severe abdominal pain as danger signs in pregnancy.

The study revealed the first action taken in response to perceived complications during pregnancy, with a high proportion of respondents using the hospital/healthcare, while others reported praying, informing their husbands, visiting the patient medicine store, taking herbal concoction and contacting friends and family as their first action. The findings contrast the findings of Egbuniwe et al. [19], who discovered that the majority of the respondents informed their husbands as the first action in response to perceived obstacles; this could be related to the study's geographical diversity. According to the study's findings, pregnant women in the community prefer to seek healthcare at PHCs, teaching hospitals, religious homes and traditional birth attendants. Some of these women do not rely solely on the PHCs for their ANC but, in addition, register in other healthcare facilities to boost their access to healthcare in pregnancy. This indicates a positive trend in ANC attendance among the respondents, suggesting a shift away from home deliveries, which could otherwise increase the risk of maternal deaths, particularly in cases of complications. The registration in multiple healthcare facilities could be due to some challenges they might have encountered in the course of their attendance at these PHCs which may include distance to the PHCs, transportation challenges and perceived quality of care, which can deter them from seeking timely and appropriate medical assistance. This implies that they were willing to attend ANC visits to safeguard their optimal health and their unborn infants. It is thus expected that this will reduce the risk of morbidity and mortality due to poor healthcare-seeking practices and limited access to health facilities among pregnant women.

This is similar to the findings of Egbuniwe et al. [19], who discovered that pregnant women seek care from primary health facilities, traditional birth attendance centres, religious homes and hospitals. The findings are similar to the findings of a study conducted by Gopalakrishnan et al. [5] on health-seeking behaviour among antenatal and postnatal rural women in Tamil Nadu's Kancheepuram District, which revealed that women seek care and delivery services from PHCs, district hospitals and tertiary hospitals. However, Peprah et al. [25] found that in rural Ghana, traditional midwives dominate childbirth care, with modern healthcare sought only in cases of severe complications.

According to more than half of the respondents, the husband's decision, the distance between healthcare centres, the facilities at the centres and the type of personnel working at the centres are the primary factors influencing healthcare-seeking behaviour during pregnancy. This is consistent with the findings of a study conducted by Mwilike et al. [9], who discovered that the perceived severity of the ailment, distance to the health institution and financial position all have a significant impact on women's decision to seek care. However, respondents were observed to rely on more than one healthcare facility to seek care, due to constraints affecting the use of PHCs, such as distance, facilities at the PHC, health personnel and quality of care which corroborate with the claim of Osuala et al. [26] which stated that pregnant women visit different healthcare centres based on the perceived seeking benefits and factors, such as facilities, accessibility and attitudes of the workers. PHCs are expected to be close to the people; however, this is not the case in some parts of Nigeria. Sometimes, the PHCs are not located within ease of proximity to the members of the community. Several women had to travel long distances to get access to these health facilities, and in situations where they are located within their locality, the facilities available are inadequate with a limited number of healthcare personnel who often visit occasionally and are not permanent staff of the facility; hence, women travel several miles to access PHCs with adequate facilities and health personnel to meet their needs. This is also responsible for the use of more than one healthcare facilities by these women to have an alternative in the event of the PHCs not meeting their expectations. Some of the PHCs in the study area are located in the rural part of the LGA of study, which may have a limited number of health facilities and healthcare personnels; thus, they rely on more than one healthcare facilities as seen in this study.

Education and spousal decision-making also play significant roles in healthcare choices across regions. Low educational attainment has been reported to significantly influence the health-seeking behaviours of pregnant women in Nigeria, impacting their utilization of healthcare services [27]. Studies indicated that women with lower levels of education are less likely to attend ANC, utilize maternal healthcare services and seek help for pregnancy-related complications [28]. In Northern Nigeria, the lack of educational opportunities for women and the male-dominated decision-making process negatively impact healthcare-seeking behaviours [27]. However, in this study, there was no significant association between the education level of the respondents and their healthcare-seeking behaviour. This may be attributed to the location of the PHCs, offering affordable services which makes them accessible to the women irrespective of their educational attainment. Also, community health promotion services delivered through channels, such as radio, television and other outreach programmes, may be responsible for disseminating maternal health information to women regardless of their education level.

In the study, more than half of the respondents stated that their husbands were the primary decision-makers when it came to seeking healthcare. This is in correlation with the findings from Northern Nigeria, where patriarchal structures often limit women's autonomy in healthcare decision-making [29]. Male dominance in healthcare decisions, especially in rural areas, has been shown to cause delays in seeking necessary medical attention, contributing to maternal morbidity and mortality, and engaging men in maternal health programmes, as suggested by Hassan and Basirka [27], could significantly improve healthcare-seeking behaviours and reduce these delays.

## 5. Conclusion

The study reveals that sociodemographic characteristics, such as age, religion, occupation, distance to healthcare centres and services rendered by healthcare personnel, significantly influence pregnant women's healthcare-seeking behaviours. Most pregnant women are aware of pregnancy risk indicators and seek medical attention if they have any issues. They rely on healthcare personnel for information and seek formal healthcare services. However, the study found no correlation between educational level and healthcare-seeking behaviours among pregnant women, suggesting that education does not significantly influence these behaviours. Overall, these findings highlight the importance of understanding and addressing healthcare needs among pregnant women.

A public health campaign is recommended to encourage healthcare-seeking behaviour among pregnant women. The public health system should be strengthened, with medical professionals providing information on early prenatal care and healthy pregnancy practices. Government and policymakers should create programmes to help overcome obstacles, such as distance and facility costs.

## Figures and Tables

**Table 1:** Sociodemographic and socioeconomic characteristics of the respondents.

Variables	Frequency	Percentage (%)
*Age (yrs.)*		
18–23	53	26.5
24–29	90	45.0
30–34	33	16.5
35–39	18	9.0
40–44	5	2.5
45–49	1	0.5
Mean ± S.D	27.03 ± 5.58 years	

*Marital status*		
Married	188	94.0
Single	9	4.5
Divorced/separated	1	0.5
Widow	2	1.0

*Gestational age*		
First trimester	62	31.0
Second trimester	83	41.5
Third trimester	55	27.5

*Religion*		
Christianity	124	62.0
Muslim	71	35.5
Traditional	5	2.5

*Occupation*		
Trader/businesswoman	89	44.5
Civil servant	37	18.5
Farmer	8	4.0
Banker	5	2.5
Housewife/unemployed	15	7.5

*Education level*		
Primary	11	5.5
Secondary	97	48.5
Tertiary	85	42.5
No formal education	7	3.5

*Occupation of spouse*		
Trader/businessman	69	34.5
Civil servant	43	21.5
Banker	8	4.0
Engineer	21	10.5
Healthcare workers	7	3.5
Farmer	17	8.5
Artisan	24	12.0
Unemployed	2	1.0

*Type of family*		
Monogamy	179	89.5
Polygamy	21	10.5
Total	200	100.0

**Table 2:** Maternal health and antenatal care indicators among the respondents.

Variables	Frequency (*N*)	Percent (%)
*Is the pregnancy planned?*		
Yes	149	74.5
No	51	25.5

*At what stage did antenatal start?*		
First trimester	114	57.0
Second trimester	76	38.0
Third trimester	10	5.0

*Have you had any stillbirths/miscarriages?*		
Yes	28	14.0
No	172	86.0

*How many stillbirths/miscarriage?*		
1	14	7.0
2	13	6.5
3	1	0.5
None	172	86.0

*Do you have any health challenges or medical conditions?*		
Yes	16	8.0
No	184	92.0

*Danger signs during pregnancy can cause a problem.*		
Yes	157	78.5
No	43	21.5

*Where did you get knowledge of danger signs?*		
Health/medical personnel	150	75.0
Social media	19	9.5
Friends	7	3.5
Relatives/family	24	12.0
Total	200	100.0

**Table 3:** Knowledge of the danger signs of pregnancy among the respondents.

Danger signs	Yes *f* (%)	No *f* (%)	Total *f* (%)
Vaginal bleeding	137 (68.5)	63 (31.5)	200 (100)
Swelling of feet and face	137 (68.5)	63 (31.5)	200 (100)
Marked reduction in foetal movement	143 (71.5)	57 (28.5)	200 (100)
Blurred vision with high fever	139 (69.5)	61 (30.5)	200 (100)
Persistent abdominal pain	154 (77.0)	46 (23.0)	200 (100)
High fever	162 (81.0)	38 (19.0)	200 (100)
Weakness	172 (86.0)	28 (14.0)	200 (100)
Convulsions	89 (44.5)	111 (55.5)	200 (100)
Breathing difficulty	108 (54.0)	92 (46.0)	200 (100)

**Table 4:** Healthcare-seeking practices.

Variables	Frequency (*N*)	Percent (%)
*Any health problem apart from pregnancy*		
Yes	16	8.0
No	184	92.0

*Health problem/ill health*		
Asthma	3	1.5
Ulcer	8	4.0
Diabetes	1	0.5
Hypertension	1	0.5
None	187	93.5

*Do you think other health problems are related to pregnancy?*		
Yes	14	7.0
No	186	93.0

*The first action towards a perceived pregnancy complication*		
Pray	36	18.0
Visit the hospital/health centre	109	54.5
Inform husband	44	22.0
Visit a patient medicine store/chemist	3	1.5
Contact friends/family members	4	2.0
Take herbal concoction	4	2.0

*Reason for choice of primary healthcare centre*		
Religious belief	22	11.0
Family choice/husband decision	96	48.0
Proximity/distance	34	17.0
Cheap service/finance	12	6.0
Facilities at the centre	32	16.0
Type of personnel working there	4	2.0

*Who influenced your decision to PHC?*		
Husband	106	53.0
Self	68	34.0
Neighbours	4	2.0
Friends	9	4.5
Relatives	13	6.5

*Do you register in the hospital apart from PHC?*		
Yes	74	37.0
No	126	63.0

*Other healthcare used*		
Primary health centre alone	129	64.5
Religious home and PHC	11	5.5
General hospitals and PHC	49	24.5
Teaching hospitals and PHC	8	4.0
Total	200	100.0

**Table 5:** Sociodemographic factors associated with the health problem among respondents.

Variables	Health problem		Total	Chi-square	*p*-value
	Yes (%)	No (%)			
*Age*					
18–23	4 (25.0)	49 (26.6)	53 (26.5)	12.155	**0.033**
24–29	7 (43.8)	83 (45.1)	90 (45.0)		
30–34	3 (18.8)	30 (16.3)	33 (16.5)		
35–39	1 (6.3)	17 (9.2)	18 (9.0)		
40–44	0 (0.0)	5 (2.7)	5 (2.5)		
45–49	1 (6.3)	0 (0.0)	1 (0.5)		

*Marital status*					
Married	14 (87.5)	174 (94.6)	188 (94.0)	2.812	0.421
Single	2 (12.5)	7 (3.8)	9 (4.5)		
Divorced/separated	0 (0.0)	1 (0.5)	1 (0.5)		
Widow	0 (0.0)	2 (1.1)	2 (1.0)		

*Gestational age*					
First trimester	10 (62.5)	52 (28.3)	62 (31.0)	8.13	**0.017**
Second trimester	4 (25.0)	79 (42.9)	83 (41.5)		
Third trimester	2 (12.5)	53 (28.8)	55 (27.5)		

*Religion*					
Christianity	10 (62.5)	114 (62.0)	124 (62.0)	1.067	0.586
Muslim	5 (31.3)	66 (35.9)	71 (35.5)		
Traditional	1 (6.3)	4 (2.2)	5 (2.5)		

*Education level*					
Primary	2 (12.5)	9 (4.9)	11 (5.5)	2.822	0.42
Secondary	6 (37.5)	91 (49.8)	97 (48.5)		
Tertiary	8 (50.0)	77 (41.8)	85 (42.5)		
No formal education	0 (0.0)	7 (3.8)	7 (3.5)		
Total	16 (100)	184 (100)	200 (100)		

*Note:* Chi-square tests are significant at *p* < 0.05. Bold values are significant at a *p* value < 0.05.

**Table 6:** Sociodemographic characteristics and healthcare-seeking practices (reason for the choice of PHC) of the respondents.

Variables	Religious belief *N*%	Husband decision *N*%	Distance *N*%	Finance *N*%	Centre facilities *N*%	Health worker *N*%	Total *N*%	Chi-square test	*p*-value
*Marital status*									
Married	18 (81.8)	93 (97.1)	33 (97.1)	10 (83.3)	31 (96.9)	3 (75.0)	188 (94)	68.45	**0.00**
Single	4 (18.2)	2 (2.1)	1 (2.9)	1 (8.3)	1 (3.1)	0 (0.0)	9 (4.5)		
Widow	0 (0.0)	1 (1.0)	0 (0.0)	1 (8.3)	0 (0.0)	0 (0.0)	2 (1.0)		

*Occupation of spouse*									
Trader/businessman	10 (27.8)	39 (35.8)	17 (38.6)	0 (0.0)	2 (50.0)	1 (25.0)	69 (34.5)	53.747	0.72
Civil servant	9 (25.0)	27 (24.8)	6 (13.6)	1 (33.3)	0 (0.0)	0 (0.0)	43 (21.5)		
Banker	1 (2.8)	5 (4.6)	2 (4.5)	0 (0.0)	0 (0.0)	0 (0.0)	8 (4.0)		
Engineer	5 (13.9)	10 (9.2)	6 (13.6)	0 (0.0)	0 (0.0)	0 (0.0)	21 (10.5)		
Healthcare workers	2 (5.6)	3 (2.8)	2 (4.5)	0 (0.0)	0 (0.0)	0 (0.0)	7 (3.5)		
Farmer	5 (13.9)	4 (3.7)	3 (6.8)	1 (33.3)	1 (25.0)	3 (75.0)	17 (8.5)		
Artisan	1 (2.8)	15 (13.8)	7 (15.9)	0 (0.0)	1 (25.0)	0 (0.0)	24 (12.0)		
Unemployed	0 (0.0)	1 (0.9)	1 (2.3)	0 (0.0)	0 (0.0)	0 (0.0)	2 (1.0)		

*Note:* Chi-square tests are significant at *p* < 0.05. Bold values are significant at a *p* value < 0.05.

**Table 7:** Sociodemographic characteristics and the healthcare-seeking practices (healthcare-seeking influence) of the respondents.

Variables	Husband *N*%	Self *N*%	Neighbours *N*%	Friends *N*%	Relatives *N*%	Total *N*%	*χ* ^2^	*p*-value
*Marital status*								
Married	101 (95.3)	64 (94.1)	2 (50)	9 (100)	12 (92.3)	188 (94)	0.379	**0.004**
Single	3 (2.8)	4 (5.9)	2 (50)	0 (0.0)	0 (0.0)	9 (4.5)		
Widow	1 (0.9)	0 (0.0)	0 (0.0)	0 (0.0)	0 (0.0)	1 (0.5)		

*Religion*								
Christianity	61 (57.5)	46 (67.6)	1 (25)	8 (88.9)	8 (61.5)	124 (62)	44.87	**0.000**
Muslim	43 (40.6)	22 (32.4)	1 (25)	1 (11.1)	4 (30.8)	71 (35.5)		
Traditional	2 (1.9)	0 (0.0)	2 (50)	0 (0.0)	1 (7.7)	5 (2.5)		

*Occupation*								
Trader/businesswoman	49 (46.2)	33 (48.5)	1 (25.0)	4 (44.4)	2 (15.4)	89 (44.5)	43.95	**0.008**
Civil servant	2.8 (26.4)	7 (10.3)	0 (0.0)	1 (11.1)	1 (7.7)	37 (18.5)		
Banker	2 (1.9)	2 (2.9)	0 (0.0)	1 (11.1)	0 (0.0)	5 (2.5)		
Housewife/unemployed	6 (5.7)	6 (8.8)	1 (25)	0 (0.0)	2 (15.4)	15 (7.5)		
Artisan	12 (11.3)	7 (10.3)	1 (25)	2 (22.2)	7 (53.8)	29 (14.5)		
Others	6 (5.7)	9 (13.2)	0 (0.0)	1 (11.1)	1 (7.7)	17 (8.5)		

*Note:* Chi-square tests are significant at *p* < 0.05. Bold values are significant at a *p* value < 0.05.

**Table 8:** Sociodemographic and healthcare-seeking practices (report of port of call for healthcare) of the respondents.

Variables	PHC alone *N*%	PHC and religion *N*%	PHC and general hospitals *N*%	PHC and teaching hospitals *N*%	PHC and traditional birth attendants *N*%	Total *N*%	*χ* ^2^	*p*-value
*Religion*								
Christianity	82 (63.6)	7 (63.6)	30 (61.2)	3 (37.5)	2 (66.7)	124 (62)	17.824	**0.023**
Muslim	45 (34.9)	3 (27.3)	18 (36.7)	5 (62.5)	0 (0.0)	71 (35.5)		
Traditional	2 (1.6)	1 (9.1)	1 (2.0)	0 (0.0)	1 (33.3)	5 (2.5)		

*Occupation of spouse*								
Trader/businessman	42 (32.6)	6 (54.5)	16 (32.7)	3 (37.5)	2 (66.7)	69 (34.5)	50.428	**0.02**
Civil servant	26 (20.2)	3 (27.3)	13 (26.5)	0 (0.0)	1 (33.3)	43 (21.5)		
Banker	2 (1.6)	0 (0.0)	3 (6.1)	3 (37.5)	0 (0.0)	8 (4.0)		
Engineer	11 (8.5)	0 (0.0)	9 (18.4)	1 (12.5)	0 (0.0)	21 (10.5)		
Healthcare workers	6 (4.7)	0 (0.0)	0 (0.0)	1 (12.5)	0 (0.0)	7 (3.5)		
Farmer	13 (10.1)	1 (9.1)	3 (6.1)	0 (0.0)	0 (0.0)	17 (8.5)		
Artisan	19 (14.7)	0 (0.0)	5 (10.2)	0 (0.0)	0 (0.0)	24 (12)		
Unemployed	2 (1.6)	0 (0.0)	0 (0.0)	0 (0.0)	0 (0.0)	2 (1.0)		
Others	8 (88.9)	1 (11.1)	0 (0.0)	0 (0.0)	0 (0.0)	9 (100)		

*Education level*								
Primary	7 (5.4)	1 (9.1)	3 (6.1)	0 (0.0)	0 (0.0)	11 (5.5)	3.782	0.987
Secondary	63 (48.8)	5 (45.5)	24 (49)	3 (37.5)	2 (66.7)	97 (48.5)		
Tertiary	53 (41.1)	5 (45.5)	21 (42.9)	5 (62.5)	1 (33.3)	85 (42.5)		
No formal education	6 (4.7)	0 (0.0)	1 (2.0)	0 (0.0)	0 (0.0)	7 (3.5)		

*Note:* Chi-square tests are significant at *p* < 0.05. Bold values are significant at a *p* value < 0.05.

## Data Availability

All data generated or analysed during this study are included in the manuscript and its supporting information files. Raw data may be made available by the authors upon reasonable request.
